# Editorial: Sleep apnea in cardiovascular disease

**DOI:** 10.3389/frsle.2024.1383738

**Published:** 2024-03-12

**Authors:** Liliana Otero

**Affiliations:** Department of Craniofacial Systems, Pontificia Universidad Javeriana, Bogotá, Colombia

**Keywords:** sleep apnea, cardiovascular disease, artificial intelligence, biomarker, machine learning

Sleep apnea is a risk factor for cardiovascular disease (CVD). Obstructive Sleep Apnea (OSA) increases the risk for coronary artery disease, hypertension, atrial fibrillation, and cardiovascular mortality. Additionally, Central Sleep Apnea is associated with atrial fibrillation in patients with heart failure. However, the pathophysiological mechanisms involved in the development of CVD associated with sleep apnea have not been completely elucidated. Although excessive daytime sleepiness (EDS) has been proposed as a marker of CVD risk, EDS may be present in individuals without sleep disorders. Therefore, it is necessary to study the role of EDS in CVD in individuals with OSA.

Zhang et al. explored the effect of EDS, low slow-wave sleep (SWS), and short sleep duration on the risk of CVD in patients with OSA. In their study, 4,475 patients who underwent polysomnography (PSG) were classified into OSA patients and controls (snorers). EDS and low SWS were identified as risk factors for moderate to high 10-year CVD risk in the entire cohort. Although EDS is an important factor for consideration in the treatment of OSA, snoring has recently been associated with CVD and metabolic syndrome. Additionally, SWS has been studied as a risk factor for hypertension and CVD in both OSA and non-OSA patients. In contrast to several studies, the study by Zhang et al. did not find any effect of sleep duration on CVD risk. They speculated that PSG might not accurately reflect individual sleep habits. However, the association between sleep duration and CVD should consider several factors such as physical activity, lifestyle habits, comorbidities, and genetics, among others.

Heart rate is a physiological process involved in regulatory mechanisms and is associated with autonomic dysfunction in patients with CVD and those with OSA. Furthermore, heart rate variability has been proposed as a risk marker for severity in these patients, and pulse rate variability might be used as a biomarker for CVD in Sleep Disordered Breathing (SDB). The prediction of the severity of SDB was the objective of the study by Maresh et al., who developed and validated an automated algorithm using recordings from 1,438 individuals. This algorithm correlates automated respiratory-related heart rate measurements derived from pulse rate with polysomnogram recordings from 2,091 individuals. They showed that their algorithm was an accurate method to predict SDB and proposed that mathematical models might be used to diagnose SDB.

Artificial Intelligence employs machine and deep learning methods to predict CVD risk in individuals with Sleep Apnea (SA) and for healthcare diagnosis in precision medicine. Although CVD, including Coronary Artery Disease (CAD), Atrial Fibrillation (AF), and cerebrovascular disease, increases morbidity and mortality among these individuals, SA and AF are often underdiagnosed. Silva et al., utilizing the K-means clustering technique, k-nearest neighbor (kNN) algorithm, and survival analysis, analyzed the Electronic Medical Record (EMR) database of 22,302 individuals. Their objective was to evaluate the role of CAD and SA in determining the risk of AF through cluster and survival analysis and to develop a risk model for predicting AF. Their algorithm, which was developed and validated, identified AF, CAD, and SA with high accuracy and sensitivity. Furthermore, they reported that CAD, SA, chronic kidney disease, and high blood pressure are significant risk factors for developing AF in a Latin-American population.

Precision medicine includes the use of artificial intelligence and omics for diagnosis through algorithms and biomarkers. Myeloperoxidase (MPO) plays a significant role in oxidative stress and inflammation, and it has been reported as a biomarker for OSA. However, the role of this enzyme in OSA has not been elucidated. Tang et al. conducted a bidirectional two-sample Mendelian randomization (MR) study to examine the causal relationship between MPO and OSA. They evaluated 38,998 OSA cases and 336,659 controls from the *FinnGen dataset* and showed an increased risk of OSA associated with elevated MPO levels. However, a causal relationship between OSA and MPO could not be demonstrated. Additional studies are necessary to identify biomarkers that might be employed as therapeutic targets in the future.

The treatment of CVD in patients with sleep apnea is complex and involves a multidisciplinary team. OSA and Central Sleep Apnea (CSA) are common in patients with heart failure and contribute to increased hospital readmission rates and mortality risk in these patients. The first therapeutic option for this type of patient is to optimize cardiac function. In this Research Topic, Mashaqi et al. report a case of a patient with severe heart failure, refractory ventricular arrhythmia, OSA, and CSA who had a complete resolution of both OSA and CSA 60 days post-Left Ventricular Assist Device (LVAD) implantation. They describe some pathophysiological mechanisms involved in these comorbidities and in the prognostic success of LVAD for OSA and CSA treatment in patients with heart failure.

This Research Topic includes three studies that propose new markers for the diagnosis and prognosis of CVD in patients with SA. These markers have been identified through cross-sectional studies and artificial intelligence methods. Another study proposes a biomarker based on the pathophysiology of SA, and the case report article describes the resolution of SA through cardiac treatment. The future frontiers of research in this topic should be directed toward identifying new biomarkers for diagnosis and prognosis, as well as therapeutic targets based on the findings of artificial intelligence and precision medicine for CVD and SA.

## Author contributions

LO: Writing—review & editing.

